# Prevalence and Public-health Significance of HIV Infection and Anaemia among Pregnant Women Attending Antenatal Clinics in South-eastern Nigeria

**Published:** 2007-09

**Authors:** C.J. Uneke, D.D. Duhlinska, E.B. Igbinedion

**Affiliations:** 1 Department of Medical Microbiology/Parasitology, Faculty of Clinical Medicine, Ebonyi State University, Abakaliki, Nigeria; 2 Department of Zoology, Faculty of Natural Sciences, University of Jos, Nigeria; 3 Department of Applied Microbiology, Faculty of Applied and Natural Sciences, Ebonyi State University, Abakaliki, Nigeria

**Keywords:** Anaemia, Cross-sectional surveys, HIV, HIV infections, Maternal health, Morbidity, Mortality, Pregnancy, Pregnancy outcomes, Prevalence, Risk factors, Nigeria

## Abstract

HIV infection and anaemia are major public-health problems in Africa and are important factors associated with an increased risk of adverse pregnancy outcomes. The objective of this study was to determine the prevalence of HIV infection and anaemia among pregnant women attending antenatal clinics in south-eastern Nigeria. To achieve this, a cross-sectional survey was conducted during July 2005–June 2006 using standard techniques. Of 815 pregnant women studied, 31 (3.8%, 95% confidence interval [CI] 2.5–5.1) were HIV-positive. Maternal age and gestational age were not associated with HIV infection (p>0.05). The prevalence of anaemia (Hb<11.0 g/dL) was 76.9%, and 15 (1.8%, 95% CI 0.9–2.7) had severe anaemia (Hb<7.0 g/dL). A significantly higher prevalence of anaemia was observed among individuals in their second pregnancy trimester (p<0.05) and those infected with HIV (p<0.05). Since HIV and anaemia are preventable, antenatal care services could serve as a pivotal entry point for simultaneous delivery of interventions for the prevention and control of HIV infection and anaemia in pregnant women.

## INTRODUCTION

Despite the considerable improvement in healthcare-delivery services in many parts of the African continent, making motherhood safer, which is an urgent priority and one of several child-survival strategies applied through antenatal care, continues to be particularly challenging. Anaemia in pregnancy has been described as one of such enormous medical challenges because it is a major public-health problem in Africa and is an important factor associated with an increased risk of poor pregnancy outcomes (1) and maternal morbidity and mortality in developing countries (2). In sub-Saharan Africa, a high prevalence (95.4%) of anaemia has been reported among pregnant women (3), with the mean prevalence of maternal anaemia in the subregion estimated to be 61% (4). Anaemia even when mild is associated with reduced productivity at work (5). During pregnancy, severe anaemia may result in circulatory changes that are associated with an increased risk of heart failure (6). During labour, women with severe anaemia are less able to endure even moderate blood loss and, as a consequence, are at a higher risk of requiring a blood transfusion during delivery (7,8). In addition, severe anaemia in pregnancy is an important direct and indirect cause of maternal death (9,10), and for the foetus, severe maternal anaemia may result in intrauterine growth retardation, stillbirth, and low birthweight (11-13).

The aetiology of anaemia in pregnancy in sub-Saharan Africa is complex and multifactorial (7). Apart from malaria, other causes of anaemia in pregnancy include an iron- and folate-deficient diet and infections, such as hookworm, and increasingly human immunodeficiency virus (HIV) (14). It has been suggested that infection with HIV during pregnancy may be associated with an increased risk of anaemia-related maternal death in developing-country settings due to the increasing severity of anaemia or due to the combined effects of anaemia and other infections (15). Anaemia also has been associated with progression of HIV disease (16). Concerning the global HIV epidemic, sub-Saharan Africa remains by far the worst-affected region with 25.4 million people living with HIV (just under two-thirds, i.e. 64% of all people living with HIV) (17). The HIV/AIDS epidemic is affecting females most severely in the subregion, and women of reproductive age make up almost 57% of adults living with HIV, accounting for up to 80% of HIV-infected women in the world (17,18), with HIV-prevalence rates sometimes exceeding 40% among pregnant women (19,20).

The HIV/AIDS epidemic intersects with the problem of maternal mortality in many circumstances in sub-Saharan Africa. The extent of the contribution of HIV/AIDS to maternal mortality is difficult to quantify, as the HIV status of pregnant women in the subregion is not always known (21). HIV impacts on direct (obstetrical) causes of maternal mortality by an associated increase in pregnancy-related complications, such as anaemia, postpartum haemorrhage, and puerperal sepsis (8,21). Although anaemia is highly prevalent during pregnancy and is common during HIV infection, anaemia and iron status have not been well-characterized in HIV-infected pregnant women in most parts of sub-Saharan Africa (22). A relationship between HIV seropositivity and a decreased haemoglobin (Hb) concentration in pregnancy has been suggested, and the inclusion of HIV screening in differential diagnosis of anaemia is recommended (23). However, the paucity of comprehensive population-based data on anaemia and HIV infection in pregnancy in Nigeria and other parts of West Africa with a similar setting makes the formulation of policies that would transform into effective and sustainable intervention/control programmes for pregnant women an enormous challenge to health policy-makers.

This present study was, therefore, designed to provide scientific information on the prevalence of anaemia and HIV infection and associated factors to maternal anaemia, which can be used for directing policy development and control-programme implementation and also provide a baseline measurement on which the impact of interventions can be evaluated. The public-health implications of findings as they affect the overall maternal well-being in this part of the globe are discussed.

## MATERIALS AND METHODS

### Study area

This investigation was conducted in two major cities—Abakaliki, the Ebonyi State capital, and Enugu, the Enugu State capital—in south-eastern Nigeria during July 2005–June 2006. The study settings are separated by a distance of about 100 km and have similar socioeconomic, ethnic and religious characteristics, except that Enugu is a much more developed city than Abakaliki, particularly in terms of commerce and industries. Two of the largest hospitals in the cities—Ebonyi State University Teaching Hospital (EBSUTH), Abakaliki and Balm of Gilead Hospital (BGH), Enugu, which serve as referral centres for gynaecological services and run the biggest antenatal clinics, were used for the study.

### Ethical considerations

Ethical approval for this study was obtained from the Faculty of Clinical Medicine (Infectious Diseases Research Division, Department of Medical Microbiology), Ebonyi State University, Abakaliki, and from the ethical committee of the EBSUTH, Abakaliki and of the BGH, Enugu. Approval was granted subject to patient anonymity being maintained, good laboratory practice/quality control being ensured, and every finding being treated with utmost confidentiality and for the purpose of this research only. All work was performed according to the international guidelines for human experimentation in clinical research (24).

### Study population/sampling technique

Pregnant women in their various pregnancy trimesters who visited the antenatal clinics (ANCs) of the EBSUTH, Abakaliki, and the BGH, Enugu, within the study period, at their first visit to the antenatal care clinic since the commencement of pregnancy were considered for the study. All the subjects were verbally notified prior to sample collection, and their informed consent was duly obtained. Age of each woman and pregnancy trimester was determined by interview. A sample of about four mL of blood was obtained by venepuncture from each patient for analysis. For the purpose of the research, no personal identifiers (names, ID number, address, etc.) were used on the blood sample of the participants. Instead, bar-coded numbers were used for ensuring anonymity of the donors, facilitating laboratory procedures, and minimizing the chances of errors during the handling of the blood specimens. All specimens were analyzed within one hour of collection.

### Laboratory analysis

Blood samples obtained from the subjects at the BGH, Enugu, were analyzed at the laboratory unit of the hospital for haemoglobin (Hb) concentration using the cyanmethaemoglobin method described previously (25); reading was done using a spectrophotometer (Bayer RA 50). The packed cell volume (PCV) was determined for samples obtained from subjects at the EBSUTH, Abakaliki, using the haematocrit technique (25), at the Research Laboratory of the Department of Medical Microbiology, Ebonyi State University, Abakaliki. The PCV (%) values were converted to Hb (g/dL) by applying the constant factor of 0.3 (26). The commercially-available HIV Tri Line Test-kits (Biosystem Inc, Austria) were first used for screening the serum sample of each subject which was separated from blood to detect antibodies to HIV-1 and HIV-2. Thereafter, HIV-seropositive samples were confirmed by immunoblot analysis using the commercially-available BIORAD New Lav Blot-kits (Bio-Rad Novapath Diagnostic Group, USA). The instructions of the manufacturer were strictly followed to determine the serostatus of the samples.

Results of both HIV and haemoglobin tests were made available to all the study participants. All the pregnant women who were HIV-positive were informed of their HIV status by their physicians and were offered counselling and also enrolled into the free maternal antiretroviral therapy programme of the EBSUTH. Also, all the women after enrollment at the antenatal clinics of the two hospitals were given chemoprophylaxis, including haematinics, to treat anaemia, particularly for those who were anaemic.

### Statistical analysis

The 95% confidence interval was calculated using the standard method. The differences in proportion were evaluated using the chi-square test. The statistical significance was achieved if p value was <0.05.

## RESULTS

In this investigation, a combined total of 815 pregnant women were studied, and 31 (3.8%, 95% CI 2.5–5.1) of these women were HIV-positive. A higher prevalence of HIV infection was recorded at the EBSUTH, Abakaliki, where 21 (5.3%, 95% CI 3.1–7.5) of 394 individuals screened were infected with HIV, while at the BGH, Enugu, 10 (2.3%, 95% CI 0.9–3.7) of 421 pregnant women screened had HIV infection (Table 1). When maternal age was associated with HIV infection, the prevalence decreased with increase in age. Although individuals in the 20 years and less age-category recorded the highest prevalence of HIV infection, the difference in the trend was not statistically significant (x^2^=2.23, p>0.05). The association of HIV infection with gestational age indicated that the highest infected individuals were more likely to be in their third pregnancy trimester. However, there was also no statistically significant difference in the trend (x^2^=1.71, p>0.05) (Table 1).

**Table 1:** Prevalence of HIV infection in relation to age and gestational age among pregnant women attending antenatal care clinics in south-eastern Nigeria

	EBSUTH, Abakaliki				BGH, Enugu				Total overall			
Parameter	No. examined	HIV+ve		95% CI	No. examined	HIV+ve		95% CI	No. examined	HIV+ve		95% CI
		No.	%			No.	%			No.	%	
Age (years)												
<20	48	3	6.3	0.6–13.2	35	1	2.9	2.7–8.5	83	4	4.8	0.2–9.4
21–30	268	15	5.6	2.8–8.4	273	8	2.9	0.9–4.9	541	23	4.3	2.6–6.0
31–40	78	3	3.8	1.6–6.0	94	1	1.1	1.0–3.2	172	4	2.3	0.1–4.5
>41	0	0	0.0	-	19	0	0.0	-	19	0	0.0	-
Total	394	21	5.3	3.1–7.5	421	10	2.3	0.9–3.7	815	31	3.8	2.5–5.1
Trimester												
First	16	0	0.0	-	74	2	2.7	1.0–6.4	90	2	2.2	0.8–5.2
Second	138	7	5.1	1.4–8.8	178	3	1.7	0.2–3.6	316	10	3.2	1.3–5.1
Third	240	14	5.8	2.8–8.8	169	5	3.0	0.4–5.6	409	19	4.6	2.6–6.6
Total	394	21	5.3	3.1–7.5	421	10	2.3	0.9–3.7	815	31	3.8	2.5–5.1

BGH=Balm of Gilead Hospital, Enugu; CI=Confidence interval; EBSUTH=Ebonyi State University Teaching Hospital, Abakaliki

Of the 815 women screened, 15 (1.8%, 95% CI 0.9–2.7) had severe anaemia (Hb <7.0 g/dL). The combined prevalence of anaemia (Hb <11.0 g/dL) was 76.9%. A higher prevalence (83.8%) of anaemia was recorded among the subjects at the EBSUTH, Abakaliki, than their counterparts at the BGH, Enugu (70.5%). The prevalence of anaemia decreased with age but the difference was not statistically significant (x^2^=5.06, p>0.05) (Table 2). When gestational age was related with anaemia, individuals in their second pregnancy trimester had a significantly higher prevalence of anaemia than those in their first and third pregnancy trimesters (x^2^=9.24, p<0.05). The prevalence of anaemia was significantly higher among the HIV-positive women than those who were HIV-negative (x^2^=7.23, p<0.05) (Table 2). The mean haemoglobin concentration of the HIV-positive women was 8.6 g/dL, while that of the HIV-negative women was 10.2 g/dL.

**Table 2:** Prevalence of anaemia in relation to age, gestational age, and HIV infection among pregnant women attending antenatal clinics in south-eastern Nigeria

	EBSUTH, Abakaliki				BGH, Enugu				Total overall			
Parameter	No. examined	Hb <11.0 g/dL		95% CI	No. examined	Hb <11.0 g/dL		95% CI	No. examined	Hb <11.0 g/dL		95% CI
		No.	%			No.	%			No.	%	
Age (years)												
<20	48	43	89.6	81.0–98.2	35	27	77.1	63.2–91.0	83	70	84.3	76.5–92.1
21–30	268	225	84.0	79.6–88.4	273	194	71.1	65.7–76.5	541	419	77.4	73.9–80.9
31–40	78	62	79.5	70.5–88.5	94	63	67.0	57.5–76.6	172	125	72.7	66.0–79.4
<41	0	0	0.0	-	19	13	68.4	47.5–89.3	19	13	68.4	47.5–89.3
Total	394	330	83.8	80.2–87.4	421	297	70.5	66.1–74.9	815	627	76.9	74.0–79.8
Trimester												
First	16	12	75.0	54.8–96.2	74	52	70.3	59.9–80.7	90	64	71.1	61.7–80.5
Second	138	124	89.9	84.9–94.9	178	137	77.0	70.8–83.2	316	261	82.6	78.4–86.8
Third	240	194	80.8	75.8–85.8	169	108	63.9	56.7–71.4	409	302	73.8	69.5–78.1
Total	394	330	83.8	80.2–87.4	421	297	70.5	66.1–74.9	815	627	76.9	74.0–79.8
HIV infection												
HIV positive	21	21	100.0	-	10	9	90.0	71.4–100.0	31	30	96.8	90.6–100.0
HIV negative	373	309	82.8	79.0–86.6	411	288	70.1	65.7–74.5	784	597	76.1	73.1–79.1
Total	394	330	83.8	80.2–87.4	421	297	70.5	66.1–74.9	815	627	76.9	74.0–79.8

BGH=Balm of Gilead Hospital, Enugu; CI=Confidence interval; EBSUTH=Ebonyi State University Teaching Hospital, Abakaliki; Hb=Haemoglobin concentration

## DISCUSSION

The results of this investigation confirmed the results of earlier reports from various parts of sub-Saharan Africa which indicated that the prevalence of anaemia among pregnant women is unacceptably high in the region (3-7). Although anaemia in pregnancy in sub-Saharan Africa has multiple aetio-logies, in countries with a high prevalence of HIV infection, anaemia due to underlying nutritional deficiencies may be intensified by parasitic infections, compromised immunity, and the haematological consequences of chronic and systemic inflammation (14). In this present investigation, up to 76.9% of the total number of pregnant women studied were anaemic (with Hb <11 g/dL), and HIV infection was a major contributor to anaemia seen in pregnancy. These findings are consistent with those of some earlier reports (8,15,16,22). A younger age appeared to be a higher risk factor to both HIV infection and anaemia but the differences were not statistically significant (p>0.05).

The relationship between HIV seropositivity and a decreased haemoglobin concentration in pregnancy, which was suggested earlier (23), was confirmed in the present study. Our results showed that the prevalence of anaemia was significantly higher among the HIV-positive women than among the HIV-negative women (p<0.05), and a lower mean haemoglobin concentration was recorded among the HIV-positive women compared to the HIV-negative women. This was consistent with the findings of similar studies in Kisumu, Kenya (27), Harare, Zimbabwe (28), Blantyre, Malawi (22,29), and Bobo-Dioulasso, Burkina Faso (31). The underlying mechanisms by which HIV infection produces anaemia are not fully understood. However, the suggested mechanisms include a direct effect of the virus itself (32), bone marrow suppression as a result of cytokine release (33), anaemia as a result of chronic inflammation or opportunistic infection (34), reduced erythropoietin production and response (35), and deficiency of vitamin B12 or folates (29). The implication of these findings is that infection with HIV should be considered a possible additional aetiologic factor having a significant effect on haemoglobin levels among pregnant women even in the absence of evidence of concurrent opportunistic infection and/or nutritional deficiency. An earlier report suggested that, in areas with a high prevalence of HIV positivity, studies pertaining to anaemia and its aetiology should, therefore, include an assessment of HIV status (29).

It is pertinent to note that the apparently high rate of anaemia recorded among the HIV-negative women (76.1%) in this study clearly showed that HIV infection was not the only major determinant of anaemia observed among the subjects. In many areas of sub-Saharan Africa, including Nigeria, malaria and iron deficiency have been shown to substantially produce anaemia in pregnancy (3,7,11). In a review of studies of *Plasmodium falciparum*-related anaemia in pregnant women, it was suggested that up to 400,000 pregnant women develop moderate or severe anaemia (Hb <8.0 g/dL or haematocrit <25%) each year in sub-Saharan Africa as a result of malaria infection (30). Thus, in populations with a high risk of exposure to infectious diseases, particularly malaria, the vicious cycle of infection, impaired immunity, and anaemia may result in a stronger association between HIV infection and anaemia at earlier stages of the disease (14). Iron deficiency has been described as the most common cause of anaemia relating to nutrition (1). Other nutritional deficiencies (vitamin B12, folic acid, and vitamin A), congenital blood disease, alpha-thala-ssaemia, and glucose-6-phosphate deficiency) and some infections (hookworm, schistosomiasis, or tuberculosis) may contribute to anaemia in pregnancy (3,4,6,7). Iron-deficiency anaemia has, however, assumed a major maternal health problem in the subregion mainly because many people cannot afford foods rich in haem iron, and other dietary factors or cooking methods may inhibit absorption of iron (6). This may have had a considerable contribution to anaemia observed in this study. Although it was reported that the dietary intake of iron by people living in developing countries is generally high (36), iron deficiency remains prevalent in most developing countries, including Nigeria (37). This apparent paradox is because the iron being consumed is predominantly in the non-haem form, which is poorly absorbed, and some of this non-haem iron is from contamination of food with iron from soil, dust, and water; iron leaching into food during storage and cooking; contamination during food processing, such as milling; and the practice of geophagy (14,36).

Furthermore, we observed from our study that most pregnant women did not commence the intake of chemoprophylactic drugs, including haematinics and folate, until they commenced to attend antenatal care clinics. It was, therefore, not surprising that the majority of the participants in this study was anaemic as they were on their first visit to antenatal care clinics since the commencement of their present pregnancy and had not started taking routine pregnancy drugs. Reports from various parts of sub-Saharan Africa have consistently indicated that women attending the antenatal care clinic have a high prevalence of anaemia (6,7). The findings of this study, therefore, suggest that antenatal measures that can successfully target this problem are of critical importance. Measures, such as routine chemoprophylaxis which is usually administered during visit to antenatal care clinic as pregnancy progresses, have been found to be useful in reducing anaemia (4).

When gestational age was related to anaemia, interestingly, women in their second pregnancy trimester were significantly more anaemic than their counterparts in their first and third trimesters (p<0.05). The reason for this outcome was not apparently clear. However, anaemia in many areas of Africa was described as usually most severe in the second trimester of gestation, especially following a period of acute infection, e.g. malaria, in the first trimester (11,13).

Our inability to assess other possible factors that may contribute to anaemia during pregnancy in the present study population was a major drawback to this investigation. The lack of any iron indicator, i.e. ferritin or serum transferrin receptor, is a major limitation of this study. Another limitation of this study was the lack of information on the disease stage of HIV-infected women. These limitations may have affected the adequate assessment of the contributory role of HIV infection on anaemia observed among the subjects. Future research incorporating these aspects is advocated. This is because despite multiple aetiologies of anaemia in pregnancy, they are all preventable. Therefore, the knowledge of the relative importance of the different causes of anaemia in pregnancy should form the basis for intervention strategies to control anaemia (3).

Since maternal and child-health services are the most accessible health services in many communities of the developing world, antenatal care services in particular could serve as the pivotal entry point for simultaneous delivery of interventions for the prevention and control of anaemia and HIV infection in pregnant women and their neonates with linkages to the community, child health, HIV counselling and testing, treatment, care and support services, family planning, and other services (2,8,28,29). The transmission of HIV by blood transfusion makes it more urgent to prevent anaemia and avoid the need for blood transfusions. Therefore, public-health policies aimed at increasing haemoglobin levels should continue to support programmes that provide iron supplementation. Screening and treatment for anaemia should be included in HIV care initiatives, particularly those that target women (14). The public-health significance of this cannot be overstated as the resolution of HIV-related anaemia has been shown to improve the quality of life, physical functioning, energy, and fatigue in individuals with HIV infection (38). If coherence is ensured at each level of the maternal healthcare-delivery system in sub-Saharan Africa, factors associated with anaemia which, of course, are largely preventable, and the effects of anaemia on maternal health and pregnancy outcomes among HIV-infected and uninfected women could be minimized with the use of cost-effective and technologically-feasible public-health interventions.
